# RNAi Screen of DAF-16/FOXO Target Genes in *C. elegans* Links Pathogenesis and Dauer Formation

**DOI:** 10.1371/journal.pone.0015902

**Published:** 2010-12-31

**Authors:** Victor L. Jensen, Karina T. Simonsen, Yu-Hui Lee, Donha Park, Donald L. Riddle

**Affiliations:** 1 Department of Medical Genetics, University of British Columbia, Vancouver, British Columbia, Canada; 2 Michael Smith Laboratories, University of British Columbia, Vancouver, British Columbia, Canada; 3 Department of Biochemistry and Molecular Biology, University of Southern Denmark, Odense, Denmark; Duke University Medical Center, United States of America

## Abstract

The DAF-16/FOXO transcription factor is the major downstream output of the insulin/IGF1R signaling pathway controlling *C. elegans* dauer larva development and aging. To identify novel downstream genes affecting dauer formation, we used RNAi to screen candidate genes previously identified to be regulated by DAF-16. We used a sensitized genetic background [*eri-1(mg366); sdf-9(m708)*], which enhances both RNAi efficiency and constitutive dauer formation (Daf-c). Among 513 RNAi clones screened, 21 displayed a synthetic Daf-c (SynDaf) phenotype with *sdf-9*. One of these genes, *srh-100*, was previously identified to be SynDaf, but twenty have not previously been associated with dauer formation. Two of the latter genes, *lys-1* and *cpr-1*, are known to participate in innate immunity and six more are predicted to do so, suggesting that the immune response may contribute to the dauer decision. Indeed, we show that two of these genes, *lys-1* and *clc-1*, are required for normal resistance to *Staphylococcus aureus*. *clc-1* is predicted to function in epithelial cohesion. Dauer formation exhibited by *daf-8(m85)*, *sdf-9(m708)*, and the wild-type N2 (at 27°C) were all enhanced by exposure to pathogenic bacteria, while not enhanced in a *daf-22(m130)* background. We conclude that knockdown of the genes required for proper pathogen resistance increases pathogenic infection, leading to increased dauer formation in our screen. We propose that dauer larva formation is a behavioral response to pathogens mediated by increased dauer pheromone production.

## Introduction

The *C. elegans* dauer larva is a facultative diapause and dispersal stage that develops in response to adverse environmental stimuli such as high temperature, high population density or limited food [1]. Mutations in genes affecting the signal transduction pathways controlling the developmental switch may result either in constitutive dauer formation in favorable environments (dauer-constitutive, or Daf-c) or a lack of dauer formation in adverse environments (dauer-defective, or Daf-d) [2]. Though there are nearly 30 identified dauer formation (*daf*) genes in *C. elegans*, there may be many more genes that have minor effects on the known pathways that are not detectable as single mutants [3]–[5]. The major pathways involved in dauer formation are the transforming growth factor β (TGF-β), insulin/insulin-like (IIS) and guanylyl cyclase pathways [6]. Transcriptional targets of the DAF-3/Smad [7], DAF-16/FOXO [8] and DAF-12 [9] transcription factors are the effectors for parallel processes that execute the dauer/non-dauer switch.

Some of the genes involved in dauer formation function within neurons, and affect neurosensory perception or neuropeptide secretion [10]–[14]. The dauer pheromone and the competing food signal both require proper sensory perception to elicit a response [15]. Genes shown to be involved in dauer formation include a guanylyl cyclase, G-proteins and genes required for proper amphid cilia formation [10], [16], [17].

Neural tissue in *C. elegans* has been previously shown to be refractory to gene expression knockdown by RNAi [18]. This effect can be reduced with mutants that affect the RNAi process including *eri-1*, a gene that encodes a siRNAase [18]. This mutant shows a weak Daf-c phenotype when treated with RNAi targeted for the strong Daf-c genes *daf-2* and *daf-19*. Here we use a strain that contains *eri-1* as a double mutant with the synthetic dauer formation (SynDaf) mutant *sdf-9*, a phosphatase-dead phosphatase [4], [19], [20]. The genetic data suggest that *sdf-9* interacts directly with the DAF-2 insulin receptor to stabilize its phosphorylated state, thereby increasing insulin signaling [20]. Although *sdf-9(m708)* has little or no Daf-c phenotype as a single mutant, it strongly enhances most Daf-c mutants, and results in a synthetic Daf-c phenotype with other genes [4], [19], [20]. The *eri-1; sdf-9* double mutant proved itself useful for assaying enhanced dauer formation resulting from gene knockdown via RNAi.

It is known that the long-lived mutant *daf-2* has increased resistance to pathogenic bacteria [21] as well as other stresses [22]. Increased pathogen resistance has been shown to be dependent on the DAF-16/FOXO transcription factor [21] and many of the DAF-16 transcriptional target genes are predicted to function in innate immunity [23], [24]. Here we describe an RNAi screen of candidate SynDaf genes (by their identity as DAF-16 transcriptional targets) that identified eight genes associated with innate immunity. This suggests that *C. elegans* uses dauer formation and subsequent dispersal as a defensive response to pathogens in the environment.

## Results

### RNAi Screen for Enhanced Dauer Formation

As proof of concept for the use of *eri-1(mg366); sdf-9(m708)* as a sensitized genetic background to detect SynDaf mutations, we tested the effect of *akt-1* RNAi on this strain. AKT-1 is involved in transmitting the signal from the DAF-2 receptor to the DAF-16/FOXO transcription factor [25]. An *akt-1* knockout has no Daf-c phenotype as a single mutant, but forms 82% dauer larvae as a double mutant with *sdf-9* [4]. The *akt-1* RNAi treatment resulted in a median constitutive dauer formation of 44% compared to 6% for the control RNAi.

For our screen, we chose genes that were putatively repressed four-fold by DAF-16 activity (in a *daf-2* background) from two microarray analyses [23], [24], as well as those identified to be direct targets by chromatin immune-precipitation [26]. We chose repressed genes because they are down regulated upon entry into dauer (when DAF-16 is active) and RNAi also represses expression. From the RNAi library [23] we obtained clones corresponding to 513 identified target genes. Sixty-nine of these genes (13%) were obtained from two of our three sources. None were found in all three. Since DAF-16 is a major regulator of dauer formation, we hypothesized that many of its target genes may have small effects on dauer formation, detectable only in a sensitized genetic background.

### 21 SynDaf Genes

For the primary screen a qualitative assessment of dauer formation was completed for each target gene. 131 of the 513 RNAi clones were judged by visual inspection to result in increased dauer formation (compared to the control), and these were kept for further assessment (for complete target list see Table S1). These included clones that appeared to have only slightly higher dauer formation. In subsequent quantitative screens we required a target gene RNAi treatment to reproduce higher dauer formation significantly (p<0.05) for three consecutive independent trials. In the three retests, we counted each population (dauer and non-dauer larvae) and compared it to the control, if a clone failed to repeat once it was deemed to be negative. Thirty-one genes remained after a first quantitative pass, twenty-three after a second and twenty-one remained after a third and final re-test. Average percent dauer formation for each of the 21 target genes is given in Table 1 (actual counts included in Table S2).

**Table 1:** Set of 21 SynDaf Genes.

Gene	Predicted Function^a^	Average Dauer Larvae Percent ± S.E.	Combined N
**Controls**			
GFP	Negative control	5.6±1.3	1926
*akt-1*	Dauer signaling kinase, positive control	43.8±11.2	1561
**Previously Identified SynDaf Gene**			
*srh-100*	Predicted olfactory G-protein coupled receptor	37.8±12.8	417
**New SynDaf Genes**			
C53A3.2	p-Nitrophenyl phosphatase (Synthetic small brood size with *daf-18*)	26.7±11.2	842
*skr-8*	skp1 protein (Regulated by DAF-12)	26.9±0.7	420
*dct-5*	zinc finger transcription factor	12.4±2.1	780
*cyp-35A3*	Cytochrome P450 CYP2 subfamily	35.1±22.9	289
C24G6.6	Flavin-containing amine oxidase	38.8±16.0	329
*lase-1*	Aminoacylase ACY1	64.1±13.7	467
*unc-84*	Transmembrane protein with a SUN domain	28.0±7.9	430
*ccb-1*	Beta subunit of dihydropyridine sensitive L-type calcium channel	24.7±4.1	631
*dct-14*	DNAk, heat shock protein	33.2±1.1	509
E02C12.8	CHK kinase like, like SRC kinase	21.0±7.6	927
F59B1.2	Gene	23.7±8.4	708
F44D12.8	SRRM1 (serine arginine repeat nuclear matrix protein)	33.5±13.9	560
**Innate Immunity Related New SynDaf Genes**			
F35E12.9	CUB domain	31.5±9.7	276
F35E12.10^b^	CUB domain	22.0±12.3	645
ZK896.5^b^	CUB domain	35.4±10.7	436
*dct-17* b	CUB domain and inorganic phosphatase	18.5±4.9	769
*clc-1* b	Claudin	47.4±7.0	378
*lys-1* b	Lysozyme	19.1±6.5	565
*cpr-1* b	Cysteine proteinase, cathepsin L	31.8±3.8	433
F52E1.5^b^	Homology to chondroitin proteoglycan	12.5±4.0	842

Actual counts and p-values listed in Table S2.

a Predicted functions are based on previous research and Wormbase annotations.

b Upregulated upon infection [38], [39].

Whereas 69 of the 513 candidate target genes were found in two of the three sources, [23], [24], [26], eight of the 21 positives were among these 69. The probability that this was random had a p-value (χ^2^ test) of 0.001. Hence, genes from multiple sources were enriched among the 21 positives (Table 1). Nevertheless, most of the positives originated from only one of the three sources.

Each of the three source studies [23], [24], [26] identified gene classes that were enriched in each of their own data sets. The most enriched protein domain in both the 21 positives we report (Table 1) and the 513 target genes are the CUB (or CUB-like) domain (C1r/C1s, Uegf, Bmp1) [27]. It has been suggested that CUB-domain proteins function in innate immunity due to the organization of their genes in large clusters, the similarity of CUB domains to immunoglobulins and their localization at the cell surface [28], [29]. In addition, a CUB domain protein has been identified in a recent RNAi screen for sensitivity to *Pseudomonas auruginosa* PA14 infection and arsenic stress [30].

### Genes Known to Affect Dauer Formation or Insulin Secretion

Several genes identified in our screen function in pathways that have already been associated with dauer formation. This includes one gene that has already been identified as SynDaf, *srh-100* [26], [31]. SRH-100 is a predicted olfactory G-protein coupled receptor (GPCR) [32]. Detection of this gene shows that our screen can replicate previous results.

A previously unreported SynDaf gene, *ccb-1*, encodes the β-subunit of the L-type calcium channel, a protein involved in insulin secretion in mammals [33]. Calcium signaling in *C. elegans* has been shown to affect dauer formation and insulin secretion [12]. It is likely that loss of *ccb-1* results in lower insulin output, which has been previously shown in other insulin secretion mutants to result in a SynDaf phenotype [12].

### Genes with Unknown Function

Most of the 21 SynDaf genes we identified have predicted protein domains but no assigned functions (Table 1). Five have been shown to interact with *daf* genes. C53A3.2 encodes a HAD-superfamily hydrolase and was shown to have a synthetic small brood-size phenotype with *daf-18*/PTEN [34]. *skr-8*, a Skp1 homolog that is part of the proteasomal E3 ubiquitin ligase complex, has been shown to be regulated by DAF-12 [35] as well as DAF-16. Three genes (ZK896.5, F35E12.9 and *dct-5*) are differentially regulated in TGF-β mutants during dauer entry as measured by microarray analysis [5].

Three of the 21 genes have been previously shown to suppress the tumorous gonad phenotype of *gld-1* mutants in an RNAi screen of DAF-16 targets [36]. *dct-5* (DAF-16-controlled tumor suppressor) encodes a zinc finger transcription factor [36], *dct-14* encodes a heat shock protein possibly involved in germ cell apoptosis, and *dct-17* encodes a protein with CUB and inorganic phosphatase domains. This overlap between the *gld-1*-tumor-suppressor genes and the SynDaf positives in this study suggest that these overlapping genes could be involved in the IIS pathway.

Finally, three genes, F44D12.8, C24G6.6, and F59B1.2, were SynDaf under our conditions but they have no previously identified involvement in any biological process. F44D12.8 encodes a serine arginine repeat nuclear matrix protein (SRRM), which may function in alternative splicing or mRNA stability [37]. C24G6.6 encodes a flavin-containing amine oxidase and may function in neurotransmission. F59B1.2 encodes a protein with no known or predicted domains.

### Innate Immunity Genes

The most notable trend within our list of 21 SynDaf genes is that eight genes have a connection to innate immunity (Table 1). Four genes encode proteins that contain CUB domains and are members of large clusters of paralogs. Several genes in these clusters are induced upon infection [38], [39], [28], so we include these in our list of immunity genes that are SynDaf. Recently, it has been shown that several CUB-like genes are induced upon infection with *Yersinia pestis* [40]. A total of seven of the eight innate immunity related genes found in our screen, including three CUB domain proteins, *dct-17*, *clc-1*, *cpr-1* and *lys-1* are reported to be induced upon infection [38], [39].

**Figure 1:**
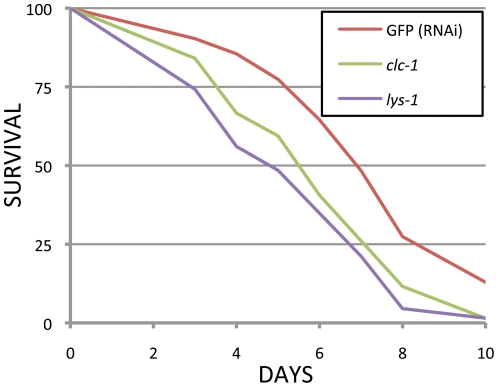
Survival of RNAi treated adults on *S. aureus*. *clc-1* and *lys-1* RNAi treatment increased pathogen sensitivity compared to the RNAi control (GFP). One of two independent tests is shown. The TD50 (time required for 50% of the nematodes to die) for *lys-1* was 4.7 days (p<0.0001) and for *clc-1* was 5.4 days (p = 0.001) compared to 6.8 days for the GFP RNAi control. p-values were calculated using the log-rank test.

To determine whether the innate immunity related positives were in fact causing sensitivity to pathogens, we tested all eight immunity genes using RNAi in the *rrf-3* RNAi hypersensitive background [41], and challenged them with *Staphylococcus aureus*. Under these conditions, two of the eight, *lys-1* and *clc-1*, had significantly reduced survival on *S. aureus* (Figure 1). Sensitivity to pathogenic bacteria has not been previously reported for either of these two genes, but LYS-1 over-expression has been shown to confer resistance to *Serratia marcescens* [42]. It is predicted that *clc-1*, which encodes a claudin-like protein, plays a role in epithelial cohesion [43]. It is possible that the epithelial layers in *C. elegans* become more permeable to *S. aureus* as a result of *clc-1* RNAi.

### Dauer Formation on Pathogenic Bacteria

To determine if pathogenesis affects dauer formation, we challenged *C. elegans* with different pathogenic bacteria. We selected the pathogens *Pseudomonas aeruginosa* PA14 [44], *S. aureus*, *Agrobacterium tumefaciens* and *S. marcescens*, all of which have been previously tested with *C. elegans* [42], [45], [46]. The strains we used reduced survival (compared to the standard laboratory food *E. coli* OP50) similarly to the previous reports (data not shown). We also used *Bacillus subtilis*, because it had been previously shown to increase the survival of *C. elegans* compared to *E. coli* OP50 [21].

We first challenged the relatively weak *daf-8(m85*ts) Daf-c mutant with all the bacterial strains at an intermediate temperature (22.5°C), except for PA14 which we tested at 15°C, a permissive temperature for *daf-8(m85)*. The percent dauer formation seen for *daf-8* increased on all three pathogens tested compared to OP50, but was reduced on *B. subtilis* (Table 2). Similarly, *sdf-9(m708)* formed ∼20% dauer larvae on OP50 (at 26°C) and 2% on *B. subtilis*, but formed more than twice as many dauer larvae on *A. tumefaciens* or *S. marcescens* and three times as many on *S. aureus* (Table 3).

**Table 2:** Percent dauer formation of *daf-8(m85)* on pathogenic bacteria at 22°C.

Bacteria	*daf-8*	N	p-value
*E. coli* OP50	65.3	101	
*B. subtilis*	12.1	107	6.3E-31
*A. tumfaciens*	84.1	195	3.7E-8
*S. marcescens*	79.4	102	2.8E-3
*E. coli* OP50^a^	0	86	
*P. aeruginosa* PA14^a^	70.6	68	5.9E-9

a These tests were carried out at 15°C.

**Table 3:** Percent dauer formation of *sdf-9(m708)* on pathogenic bacteria at 26°C.

Bacteria	*sdf-9*	N	p-value
*E. coli* OP50	19.6	97	
*B. subtilis*	2.6	76	2.0E-4
*A. tumfaciens*	49.4	79	2.6E-11
*S. marcescens*	42.2	90	6.3E-08
*S. aureus*	60.0	40	1.2E-10

We tested N2 for its response to pathogens at 27°C (a condition that induces ∼5–10% dauer larvae on OP50) to ensure the effect we observed was not unique to Daf-c mutants [11]. The same trend seen with the two weak Daf-c mutants was repeated in N2 with *A. tumefaciens* and *S. marcescens* significantly enhancing dauer formation (Table 4). We conclude that part of the *C. elegans* response to a pathogenic environment is to enter the dauer stage at greater frequency.

**Table 4:** Percent dauer formation of N2 and *daf-22(m130)* on pathogenic bacteria at 27°C.

Bacteria	N2	N	p-value^a^	*daf-22*	N	p-value^a^
*E. coli* OP50	11.7	231		3.9	246	
*B. subtilis*	0.78	129	1.1E-4	0	131	0.021
*A. tumfaciens*	27.6	98	1.0E-6	0	133	0.020
*S. marcescens*	30.6	111	5.2E-10	0	95	0.049

a p-values given are relative to OP50 sample for each genotype.

We performed epistasis analysis to determine which part of the dauer signaling pathway affects pathogenesis. We surmised that olfactory sensation might be involved because *C. elegans* is able to discriminate between bacteria [47]. To test this we used the *daf-8(m85); daf-6(e1377)* double mutant that can form dauer larvae constitutively (due to the *daf-8* mutation), but is defective in chemosensory behavior due to *daf-6* with improper formation of the sensory channel, preventing the olfactory neurons from contacting the environment [48], [49]. While the *daf-8* single mutant (which has normal olfactory behavior) responded to pathogenic bacteria by forming a higher percentage of dauer larvae (Table 2), the *daf-8;daf-6* double mutant formed fewer dauer larvae on the pathogenic bacteria (Table 5). This indicates that olfactory sensation is required for the increase in dauer formation on pathogenic bacteria.

**Table 5:** Percent dauer formation of *daf-8(m85); daf-6(e1377)* on pathogenic bacteria at 22°C.

Bacteria	*daf-8 ; daf-6*	N	p-value
*E. coli* OP50	64.5	96	
*B. subtilis*	41.1	73	2.7E-5
*A. tumfaciens*	35.7	140	9.2E-13
*S. marcescens*	27.6	105	2.4E-15

Our initial observation of increased infection causing higher dauer formation involved RNAi tests using the same bacterial strain (HT115) for control and sample. Hence, the dauer stimulus must not originate from the bacteria, but instead from the worms themselves. To test if the dauer pheromone served as an olfactory cue, we used the *daf-22(m130)* mutant that is unable to produce the pheromone [50], [51]. It has been reported that the expression of *daf-22* increases upon infection with PA14 [38]. Interestingly, *daf-22* was required for the increase in dauer formation. While a *daf-8(e1393) unc-13(e51)* strain formed more dauer larvae on pathogenic bacteria, a *daf-8(e1393) unc-13(e51); daf-22(m130)* mutant did not (Table 6). In these tests, the *unc-13* mutation (which does not affect dauer formation) served to prevent the strain from avoiding the pathogen.

**Table 6:** Percent dauer formation of *daf-8(e1393) unc-13(e51)* and *daf-8(e1393) unc-13(e51); daf-22(m130)* on pathogenic bacteria at 20°C.

Bacteria	*daf-8 unc-13*	N	p-value	*daf-8 unc-13; daf-22*	N	p-value
*E. coli* OP50	39.2	245		40.5	116	
*B. subtilis*	34.4	93	0.35	33.7	86	0.20
*A. tumfaciens*	61.9	160	4.1E-09	39.2	79	0.82
*S. marcescens*	54.5	101	0.0017	35.4	96	0.31

It was previously reported that *daf-22* mutants are able to form dauer larvae at a frequency similar to N2 at 27°C [52]. We compared *daf-22* dauer formation on pathogens at 27°C with that of N2. Whereas N2 formed more dauer larvae on the pathogenic bacteria, the *daf-22* mutant did not, forming only a few dauer larvae on the laboratory food OP50 and none on the pathogens tested (Table 4). Finally, we used the *pdaf-7*::GFP reporter gene that exhibits decreased expression with increased pheromone concentrations [53]. Indeed, GFP expression in L2 larvae decreased markedly after exposure to PA14 (Figure S1). Taken together this indicates that increased dauer pheromone production is a mechanism for increased dauer formation in response to bacterial pathogenesis.

## Discussion

Mutations in *sdf-9* have been independently isolated three times as enhancers of *unc-31*, *akt-1* and *daf-2* mutants [4], [19], [20]. Because *sdf-9* enhances the phenotype of most Daf-c mutants tested, we utilized it as a sensitized background for identifying new SynDaf genes. Of 20 previously unreported SynDaf genes, three have been shown and five are predicted to play roles in innate immunity. Five genes have been previously linked to insulin or TGF-β signaling. For example, *skr-8* is regulated by DAF-12 [35]. It is possible that some of our selected 513 target genes may not be SynDaf with *sdf-9*, similar to *akt-2* or the Eak genes [4], but still have a SynDaf phenotype with other mutants. Acknowledging this limitation, our screen nevertheless allowed for the detection of a new set of SynDaf genes and identification of a novel input into the dauer developmental decision.

It is not surprising to see an enrichment of target genes identified in two of the three sources in our positive gene set. There is not a strong consensus among the three gene sets we used [23], [24], [26], with about 13% (69/513) overlap, There are 67 genes in common between the two microarray experiments and two between the ChIP [26] and the McElwee *et al.* [23] microarray data, based on our filtering criteria. Eight of the 21 positives were present in the two microarray sources.

We designed our screen to detect genes downstream of DAF-16 that increased dauer formation as a result of reduced activity. Although *daf-2* mutants show increased pathogen resistance, our target gene set includes many down-regulated innate immunity genes. Despite down-regulation of such genes by DAF-16 the two microarray studies also include many up-regulated DAF-16 innate immunity targets [23], [24]. This explains the increased resistance of *daf-2* mutants to pathogens despite a number of down-regulated defense genes. Also, many of the innate immunity genes that are down regulated by DAF-16 in our positive gene list are up-regulated in response to other pathogens, which may represent a pathogen-specific response [38], [39].

### CCB-1 and possible feedback regulation of insulin signaling

Since *ccb-1* was detected in our screen, we conclude that its activity normally inhibits dauer formation. It encodes the β-subunit of the L-type calcium channel, which may modulate the sensitivity of the channel [33]. This gene is thought to be a direct target of DAF-16 because it was identified using DAF-16 ChIP, and its promoter contains a DAF-16 binding site [26]. It is possible that DAF-16 regulates the expression of *ccb-1* to modulate calcium signaling, which has been linked to insulin secretion in mammals and worms [12], [33]. The interaction between DAF-16 and *ccb-1* may be part of a feedback mechanism to reduce insulin secretion during the dauer development. The IIS pathway acts to inhibit DAF-16, but once DAF-16 activity reaches a critical threshold, it could antagonize insulin secretion to stabilize the dauer developmental decision.

### Germ line and *dct* genes

Three *dct* genes were found in our screen. These are putative DAF-16 targets that are *gld-1* (Germ Line Defective) tumor suppressors [36]. When *dct* expression is reduced the endomitotic tumors that grow within the germ lines of *gld-1* mutants are reduced. Germ line proliferation is actively suppressed in dauer larvae [54], so it is reasonable that the mechanisms governing cell proliferation in adults and dauer larvae may overlap. However, it is not clear why reduction of *dct* activity would trigger constitutive dauer formation in our screen. It is as though inhibition of mitotic progression (e.g., in response to starvation) feeds back to reduce TGF-β and/or insulin signaling and favor dauer arrest, but the point of feedback regulation is not known.

Other genes that regulate both germ line proliferation and dauer formation have already been identified, including AKT-1 and DAF-18/PTEN [54], [34]. In our positive gene set, C53A3.2 and *skr-8* have been previously shown to have a synthetic small brood size phenotype with *daf-18*, an indication of poor germ line proliferation [34], [55]. Although it is well known that dauer formation arrests germ line proliferation, these results suggest that the converse may also be true.

### Immunity Related Genes

Eight of the 21 positives have been previously implicated in innate immunity, four of which contain the CUB (or CUB-like) domain. The CUB domain consists of a β-barrel with similarity to immunoglobulins, and is predicted to be extracellular [38]. Three of the four CUB domain proteins found in our screen are induced by infection, as are the four remaining innate immunity genes [38], [39].

The *lys-1* lysozyme is an antimicrobial peptidoglycan *N*-acetylmuramoylhydrolase that has been shown to protect against infection in *C. elegans* [42]. The *cpr-1*, F52E1.5 and *clc-1* genes are also predicted to be protective genes because they are induced upon infection [38], [39], [56]. We propose that under the conditions of the RNAi screen, targeting of these innate immunity genes increases the animal's susceptibility to, or perception of, infection by the *E. coli* food [21], [57], [58]. As a response to this increased sensitivity to infection, the developing larvae may be predisposed to dauer dispersal. This leads to the hypothesis that it is the process of pathogenesis that stimulates increased dauer formation. Indeed, we have shown that pathogenic bacteria enhance dauer formation, and this requires the dauer pheromone.

Two genes, *lys-1* and *clc-1*, were required for normal resistance to *S. aureus*. The remaining six genes may not affect pathogenesis by *S. aureus* under our conditions for various reasons, including redundancy among gene families or pathogen-specific interactions. Over-expression of *lys-1* had been previously shown to increase survival on *S. marcescens* [42]. LYS-1 is a putative lysozyme, an antimicrobial protein, so we expected that loss of *lys-1* might make the worm sensitive to infection in spite of possible redundancy with *lys-2*. Reduction in survival has not been previously shown for *lys-1*, but the conditions and pathogens used were different [42].

*clc-1* encodes a claudin-like protein, and its expression has been seen to be induced upon infection [39], [59]. Claudins are predicted to function in epithelial cohesion, indicating that loss of *clc-1* function may cause the epithelial layer to loosen. In *C. elegans*, *clc-1* RNAi was reported to increase the permeability of the pharynx to a high molecular mass dye, TRITC-dextran [43]. Thus, increased CLC-1 in response to infection could strengthen the epithelial layers to resist pathogenesis. Indeed, we have shown that survival of *C. elegans* is significantly reduced when treated with *clc-1* RNAi followed by exposure to *S. aureus* from the L4 stage.

### Mechanism for Pathogenic Input into Dauer Formation

DAF-2 and DAF-16 have been previously linked to innate immunity. *daf-2* mutants are resistant to infection [21]. DAF-16 is required for the increased resistance of *daf-2*, just as it is for the longevity and dauer formation phenotypes [21], [60], [61]. We have shown that the production of dauer pheromone is required for pathogen induced dauer formation with the requirement of *daf-22* for the dauer induction. Olfactory sensation is also required for the increase in dauer formation, probably through the sensation of dauer pheromone. Worms infected with PA14 increase expression of *daf-22*, a gene that encodes a pheromone biosynthetic enzyme [38], [50], and by reducing expression of *daf-7*, an indicator of higher pheromone levels in the environment [53]. Taken together, the data indicate that when *C. elegans* encounters a pathogenic environment it increases pheromone production to elicit dauer formation. At higher concentrations purified components of the dauer pheromone were found to be a chemo-repellent [62] suggesting that increased dauer pheromone could deter other worms from entering the toxic environment. Pheromone deposited by 100 worms over 60 minutes decreases the response to a chemo-attractant [63].

We rule out starvation as the cause of increased dauer formation on pathogenic bacteria. It is well known that food limitation increases dauer formation [1], [64] and that *C. elegans* can display avoidance to pathogenic bacteria on plates [47], [65]. The AWB ciliated chemosensory neurons are required for this avoidance [47]. Chemosensory function is required for the pathogen induced dauer formation because no increase is observed in a chemosensory mutant, *daf-6* (Table 5). However, in the *unc-13* background we show increased dauer formation on pathogenic bacteria, which is suppressed by the *daf-22* mutation (Table 6). *daf-22* single mutants also show no increase in dauer formation on pathogenic bacteria (Table 4) despite normal chemosensory behavior [63]. This indicates that decreased nutrition is not part of the mechanism of pathogen induced dauer formation.

Animals may use cues to recognize infection in other individuals [66]. The original observations included changes in feather brightness or songs of songbirds affecting mate selection. A bird could select for those with genetic resistance to a pathogen by avoidance of potential mates that are infected [66]. Bullfrog tadpoles receive a chemical cue from infected tadpoles, and they spend less time in the presence of those tadpoles to mitigate the risk of infection [67]. We suggest that the dauer pheromone can work in a similar way, as it is used for avoidance and sexual attraction as well as dauer formation [62].

The benefit of dauer formation in a pathogenic environment could accrue from three dauer traits. First, dauer larvae do not feed, which should convey resistance to enteric infection. Second, the dauer stage is used for dispersal, permitting flight from local concentrations of pathogenic bacteria. Third, dauer larvae have a stronger cuticle [1], which could also defend against attachment or entry of pathogenic bacteria [57].

We have identified 21 SynDaf genes, each of which provides insight into dauer formation. Some genes fall into pathways and processes that have already been associated with dauer formation, whereas others suggest a new input into dauer formation, pathogenesis. Indeed, we show that pathogenic bacteria do enhance dauer formation possibly through increased pheromone production. We have explored the connection between dauer formation and suppression of germ line proliferation as well as innate immunity. Our screen is defined by the 513 putative DAF-16 target genes we used. A genome-wide screen should detect additional environmental inputs for dauer formation that do not require DAF-16.

## Methods

### Gene Target Selection and RNAi Screen

DAF-16 target genes were selected from two microarray studies, 336 from one [23] and 250 from another [24]. The targets chosen were at least four-fold down regulated in a *daf-2* background [23], [24]. An additional 87 targets were selected from a DAF-16 ChIP study [26]. Only the target genes that were in the Arhinger RNAi library were kept [68]. Bacterial cultures in the library that did not grow after three attempts were also not included, leaving a total of 513 target genes (full target list in Table S1). RNAi clone that targets *srh-100* is listed in the RNAi library as *srh-99* (or C46E10.7). An RNAi experiment that targets either *srh-100* or *srh-99* will likely knockdown both due to high nucleotide identity (88%). For simplicity, we list the target/positive in this paper as *srh-100* because it is the primary target of the RNAi experiment.

The *eri-1(mg366); sdf-9(m708)* strain was constructed by crossing *sdf-9* males with *eri-1* hermaphrodites. The double mutants were selected in the F2 generation by PCR tests for the deletion in *eri-1* and the transposon insert in *sdf-9* [18], [20]. The screen was performed by first spotting (in duplicate) 50 µl of each RNAi clone (thawed from an overnight liquid culture frozen at −80°C in 30% glycerol) onto 10 ml NG agar plates containing 100 mg/ml ampicillin and 1 mM IPTG, followed by overnight incubation at room temperature. The clones containing sequences specific for GFP and *akt-1* were used for the negative and positive controls, respectively. These controls were run with each test.

*eri-1; sdf-9* was exposed to RNAi for two generations. The two initial 60 mm plates per clone were inoculated with 2–3 L2 or L3 larvae and incubated at 20°C. On the seventh day of incubation, five F1 gravid adults were transferred to fresh RNAi plates (two per clone), made as above, and allowed to lay eggs for approximately 3 hours at room temperature. The plates were then transferred to 25.5°C, where the transferred adults become sterile [18], until the 10th day when the populations were scored for percentage dauer larvae. For the initial screen the two plates per clone were checked visually for an increase in constitutive dauer formation by comparison with the negative (GFP RNAi) and positive (*akt-1*) controls. For subsequent re-screening the dauer larvae (identified visually in the Zeiss Stemi SV 11 stereomicroscope at 660× magnification by the presence of dauer alae and radial constriction of the body) and non-dauer larvae were counted. RNAi clones were only re-tested if they produced a significant difference (p<0.05, χ^2^ test). To be kept as a positive, a clone had to show a significant difference in three independent trials with the smallest sample size >30 and largest being >100 (actual counts included in Table S2). Positive clones were confirmed by PCR using a T7 transcriptional start site primer followed by digestion by two different restriction enzymes.

### Classification and Comparison of Positives

Function was inferred from previous work, including the “Gene Summary” page on Wormbase [37], as stated for biological process enrichment assessment. Assessing GO term enrichment was completed by using the Wormbase BioMart function (WormMart) to retrieve identifiers for all genes [37], [69]. These identities were put into DAVID to identify enriched GO terms [70]. The number of genes overlapping in the target and positive gene sets were compared by a χ^2^ test and the p-value reported.

### Dauer Formation, *daf-7* Expression, and Adult Survival on Pathogenic Bacteria

Bacterial strains used were *P. aeruginosa* PA14, *A. tumefaciens* GV3101, *S. marcescens* ATCC 8100, *S. aureus* SH1000, *E. coli* HT115, and *E. coli* OP50. Fresh overnight cultures of each bacterial strain were spread on each plate to cover approximately half of the plate surface. Bacteria were not spread to the edges in order to minimize the number of dauer larvae crawling off the plate. To assay percent dauer formation, hypochlorite-purified eggs [71] were spotted on to two 60 mm plates for each bacterial strain. Dauer and non-dauer larvae were then counted as the first non-dauers reached egg-laying age. Bacterial strains were compared for nematode pathogenesis as previously described [21] by daily assay of the percent survival on each bacterial strain.

PA14 plates were made as described above for the *pdaf-7*::GFP expression analysis. L2 larvae were placed on PA14 or OP50 control plates and were assayed for GFP expression after 4 hours. GFP worms were imaged on a Zeiss Axioskop with a Qimaging Retiga 2000R camera.

For the survival assays with the eight pathogen-related RNAi treatments, *rrf-3(pk1426)* was treated with each of the RNAi expressing bacteria for two generations to maximize silencing. F2 larvae were synchronized by hypochlorite treatment followed by hatching overnight in M9 buffer. The synchronized L1's were then put on a 50-50 mixture of RNAi bacteria for the pathogen related gene and for *cdc-25.1* to sterilize them for the survival assay. L4 larvae were transferred from the RNAi plates to NG plates seeded with *S. aureus* and scored daily for survival. GraphPad Prism software was used to calculate significance using the log-rank test and to calculate the TD50 for each strain for each of two replicates. Figure 1 was created in Microsoft Excel.

## Supporting Information

Figure S1 **Reduction in *daf-7* expression on PA14.** In (A), the native GFP expression on the standard laboratory food *E. coli* OP50 from a *daf-7* promoter driving expression of GFP. The expression of *daf-7* is much reduced after are exposure to the strong pathogen PA14, as seen in (B). Images were taken with a 100× objective and 10× ocular lenses, eight hours after L2 larvae were transferred to either OP50 or PA14 from OP50 plates.
(TIF)

Table S1 **Full list of target RNAi clones.**
(DOCX)

Table S2 **Dauer and Non-dauer worm counts for positive genes.**
(DOCX)

## References

[1] Cassada RC, Russell RL (1975). The dauer larva, a post-embryonic developmental variant of the nematode *Caenorhabditis elegans*.. Dev Biol.

[2] Riddle DL, Swanson MM, Albert PS (1981). Interacting genes in nematode dauer larva formation.. Nature.

[3] Tewari M, Hu PJ, Ahn JS, Ayivi-Guedehoussou N, Vidalain PO (2004). Systematic interactome mapping and genetic perturbation analysis of a *C. elegans* TGF-beta signaling network.. Mol Cell.

[4] Hu PJ, Xu J, Ruvkun G (2006). Two membrane-associated tyrosine phosphatase homologs potentiate *C. elegans* AKT-1/PKB signaling.. PLoS Genet.

[5] Liu T, Zimmerman KK, Patterson GI (2004). Regulation of signaling genes by TGFbeta during entry into dauer diapause in *C. elegans*.. BMC Dev Biol.

[6] Jensen VL, Gallo M, Riddle DL (2006). Targets of DAF-16 involved in *Caenorhabditis elegans* adult longevity and dauer formation.. Exp Gerontol.

[7] Patterson GI, Koweek A, Wong A, Liu Y, Ruvkun G (1997). The DAF-3 Smad protein antagonizes TGF-beta-related receptor signaling in the *Caenorhabditis elegans* dauer pathway.. Gene Dev.

[8] Ogg S, Paradis S, Gottlieb S, Patterson GI, Lee L (1997). The Fork head transcription factor DAF-16 transduces insulin-like metabolic and longevity signals in *C. elegans*.. Nature.

[9] Antebi A, Yeh WH, Tait D, Hedgecock EM, Riddle DL (2000). *daf-12* encodes a nuclear receptor that regulates the dauer diapause and developmental age in *C. elegans*.. Gene Dev.

[10] Thomas JH, Birnby DA, Vowels JJ (1993). Evidence for parallel processing of sensory information controlling dauer formation in *Caenorhabditis elegans*.. Genetics.

[11] Ailion M, Thomas JH (2003). Isolation and characterization of high-temperature-induced dauer formation mutants in *Caenorhabditis elegans*.. Genetics.

[12] Speese S, Petrie M, Schuske K, Ailion M, Ann K (2007). UNC-31 (CAPS) is required for dense-core vesicle but not synaptic vesicle exocytosis in *Caenorhabditis elegans*.. J Neurosci.

[13] Ailion M, Inoue T, Weaver CI, Holdcraft RW, Thomas JH (1999). Neurosecretory control of aging in *Caenorhabditis elegans*.. Proc Natl Acad Sci.

[14] Alcedo J, Kenyon C (2004). Regulation of *C. elegans* longevity by specific gustatory and olfactory neurons.. Neuron.

[15] Golden JW, Riddle DL (1984). The *Caenorhabditis elegans* dauer larva: developmental effects of pheromone, food, and temperature.. Dev Biol.

[16] Bell LR, Stone S, Yochem J, Shaw JE, Herman RK (2006). The molecular identities of the *Caenorhabditis elegans* intraflagellar transport genes *dyf-6*, *daf-10* and *osm-1*.. Genetics.

[17] Swoboda P, Adler HT, Thomas JH (2000). The RFX-type transcription factor DAF-19 regulates sensory neuron cilium formation in *C. elegans*.. Mol Cell.

[18] Kennedy S, Wang D, Ruvkun G (2004). A conserved siRNA-degrading RNase negatively regulates RNA interference in *C. elegans*.. Nature.

[19] Ohkura K, Suzuki N, Ishihara T, Katsura I (2003). SDF-9, a protein tyrosine phosphatase-like molecule, regulates the L3/dauer developmental decision through hormonal signaling in *C. elegans*.. Development.

[20] Jensen VL, Albert PS, Riddle DL (2007). *Caenorhabditis elegans* SDF-9 enhances insulin/insulin-like signaling through interaction with DAF-2.. Genetics.

[21] Garsin DA, Villanueva JM, Begun J, Kim DH, Sifri CD (2003). Long-lived *C. elegans daf-2* mutants are resistant to bacterial pathogens.. Science.

[22] Honda Y, Honda S (1999). The *daf-2* gene network for longevity regulates oxidative stress resistance and Mn-superoxide dismutase gene expression in *Caenorhabditis elegans*.. FASEB J.

[23] McElwee J, Bubb K, Thomas JH (2003). Transcriptional outputs of the *Caenorhabditis elegans* forkhead protein DAF-16.. Aging Cell.

[24] Murphy CT, McCarroll SA, Bargmann CI, Fraser A, Kamath RS (2003). Genes that act downstream of DAF-16 to influence the lifespan of *Caenorhabditis elegans*.. Nature.

[25] Paradis S, Ruvkun G (1998). *Caenorhabditis elegans* Akt/PKB transduces insulin receptor-like signals from AGE-1 PI3 kinase to the DAF-16 transcription factor.. Gene Dev.

[26] Oh SW, Mukhopadhyay A, Dixit BL, Raha T, Green MR (2006). Identification of direct DAF-16 targets controlling longevity, metabolism and diapause by chromatin immunoprecipitation.. Nat Genet.

[27] Bork P, Beckmann G (1993). The CUB domain. A widespread module in developmentally regulated proteins.. J Mol Biol.

[28] Thomas JH (2006). Analysis of homologous gene clusters in *Caenorhabditis elegans* reveals striking regional cluster domains.. Genetics.

[29] Shivers RP, Youngman MJ, Kim DH (2008). Transcriptional responses to pathogens in *Caenorhabditis elegans*.. Curr Opin Microbiol.

[30] Nandakumar M, Tan MW (2008). Gamma-linolenic and stearidonic acids are required for basal immunity in *Caenorhabditis elegans* through their effects on p38 MAP kinase activity.. PLoS genet.

[31] Lee SS, Kennedy S, Tolonen AC, Ruvkun G (2003). DAF-16 target genes that control *C. elegans* life-span and metabolism.. Science.

[32] Robertson HM (2000). The large srh family of chemoreceptor genes in *Caenorhabditis* nematodes reveals processes of genome evolution involving large duplications and deletions and intron gains and losses.. Genome Res.

[33] Davalli AM, Biancardi E, Pollo A, Socci C, Pontiroli AE (1996). Dihydropyridine-sensitive and -insensitive voltage-operated calcium channels participate in the control of glucose-induced insulin release from human pancreatic beta cells.. J Endocrinol.

[34] Suzuki Y, Han M (2006). Genetic redundancy masks diverse functions of the tumor suppressor gene PTEN during *C. elegans* development.. Gene Dev.

[35] Shostak Y, Van Gilst MR, Antebi A, Yamamoto KR (2004). Identification of *C. elegans* DAF-12-binding sites, response elements, and target genes.. Gene Dev.

[36] Pinkston-Gosse J, Kenyon C (2007). DAF-16/FOXO targets genes that regulate tumor growth in *Caenorhabditis elegans*.. Nat Genet.

[37] Rogers A, Antoshechkin I, Bieri T, Blasiar D, Bastiani C (2008). WormBase 2007.. Nucleic Acids Res.

[38] Troemel ER, Chu SW, Reinke V, Lee SS, Ausubel FM (2006). p38 MAPK regulates expression of immune response genes and contributes to longevity in *C. elegans*.. PLoS Genet.

[39] Shapira M, Hamlin BJ, Rong J, Chen K, Ronen M (2006). A conserved role for a GATA transcription factor in regulating epithelial innate immune responses.. Proc Natl Acad Sci.

[40] Bolz DD, Tenor JL, Aballay A (2010). A conserved PMK-1/p38 MAPK is required in *Caenorhabditis elegans* tissue-specific immune response to *Yersinia pestis* infection.. J Biol Chem.

[41] Simmer F, Tijsterman M, Parrish S, Koushika SP, Nonet ML (2002). Loss of the putative RNA-directed RNA polymerase RRF-3 makes *C. elegans* hypersensitive to RNAi.. Curr Biol.

[42] Mallo GV, Kurz CL, Couillault C, Pujol N, Granjeaud S (2002). Inducible antibacterial defense system in *C. elegans*.. Curr Biol.

[43] Asano A, Asano K, Sasaki H, Furuse M, Tsukita S (2003). Claudins in *Caenorhabditis elegans*: their distribution and barrier function in the epithelium.. Curr Biol.

[44] Tan MW, Mahajan-Miklos S, Ausubel FM (1999). Killing of *Caenorhabditis elegans* by *Pseudomonas aeruginosa* used to model mammalian bacterial pathogenesis.. Proc Natl Acad Sci.

[45] Couillault C, Ewbank JJ (2002). Diverse bacteria are pathogens of *Caenorhabditis elegans*.. Infect Immun.

[46] Sifri CD, Begun J, Ausubel FM, Calderwood SB (2003). *Caenorhabditis elegans* as a Model Host for *Staphylococcus aureus* Pathogenesis.. Infect Immun.

[47] Zhang Y, Lu H, Bargmann CI (2005). Pathogenic bacteria induce aversive olfactory learning in *Caenorhabditis elegans*.. Nature.

[48] Perens EA, Shaham S (2005). C. elegans *daf-6* encodes a patched-related protein required for lumen formation.. Dev Cell.

[49] Albert PS, Brown SJ, Riddle DL (1981). Sensory control of dauer larva formation in *Caenorhabditis elegans*.. J Comp Neurol.

[50] Butcher RA, Ragains JR, Li W, Ruvkun G, Clardy J (2009). Biosynthesis of the *Caenorhabditis elegans* dauer pheromone.. Proc Natl Acad Sci.

[51] Golden JW, Riddle DL (1985). A gene affecting production of the *Caenorhabditis elegans* dauer-inducing pheromone.. Mol Gen Genet.

[52] Ailion M, Thomas JH (2000). Dauer Formation Induced by High Temperatures in *Caenorhabditis elegans*.. Genetics.

[53] Ren P, Lim C, Johnsen R, Albert PS, Pilgrim D (1996). Control of *C. elegans* Larval Development by Neuronal Expression of a TGF-beta Homolog.. Science.

[54] Narbonne P, Roy R (2006). Inhibition of germline proliferation during *C. elegans* dauer development requires PTEN, LKB1 and AMPK signalling.. Development.

[55] Sonnichsen B, Koski LB, Walsh A, Marschall P, Neumann B (2005). Full-genome RNAi profiling of early embryogenesis in *Caenorhabditis elegans*.. Nature.

[56] Wong D, Bazopoulou D, Pujol N, Tavernarakis N, Ewbank JJ (2007). Genome-wide investigation reveals pathogen-specific and shared signatures in the response of *Caenorhabditis elegans* to infection.. Genome Biol.

[57] Darby C (2005). Interactions with microbial pathogens..

[58] Garigan D, Hsu AL, Fraser AG, Kamath RS, Ahringer J (2002). Genetic analysis of tissue aging in *Caenorhabditis elegans*: a role for heat-shock factor and bacterial proliferation.. Genetics.

[59] Ren M, Feng H, Fu Y, Land M, Rubin CS (2009). Protein Kinase D Is an Essential Regulator of *C. elegans* Innate Immunity.. Immunity.

[60] Larsen PL, Albert PS, Riddle DL (1995). Genes that regulate both development and longevity in *Caenorhabditis elegans*.. Genetics.

[61] Kenyon C, Chang J, Gensch E, Rudner A, Tabtiang R (1993). A *C. elegans* mutant that lives twice as long as wild type.. Nature.

[62] Srinivasan J, Kaplan F, Ajredini R, Zachariah C, Alborn HT (2008). A blend of small molecules regulates both mating and development in *Caenorhabditis elegans*.. Nature.

[63] Matsuura T, Sato T, Shingai R (2005). Interactions between *Caenorhabditis elegans* Individuals during Chemotactic Response.. Zool Sci.

[64] Hu PJ (2007). Dauer..

[65] Pradel E, Zhang Y, Pujol N, Matsuyama T, Bargmann CI (2007). Detection and avoidance of a natural product from the pathogenic bacterium *Serratia marcescens* by *Caenorhabditis elegans*.. Proc Natl Acad Sci.

[66] Hamilton WD, Zuk M (1982). Heritable true fitness and bright birds: a role for parasites?. Science.

[67] Kiesecker JM, Skelly DK, Beard KH, Preisser E (1999). Behavioral reduction of infection risk.. Proc Natl Acad Sci.

[68] Kamath RS, Fraser AG, Dong Y, Poulin G, Durbin R (2003). Systematic functional analysis of the *Caenorhabditis elegans* genome using RNAi.. Nature.

[69] Smedley D, Haider S, Ballester B, Holland R, London D (2009). BioMart–biological queries made easy.. BMC Genomics.

[70] Dennis G, Sherman BT, Hosack DA, Yang J, Gao W (2003). DAVID: Database for Annotation, Visualization, and Integrated Discovery.. Genome Biol.

[71] Brenner S (1974). The genetics of *Caenorhabditis elegans*.. Genetics.

