# Supplementary figures and images for: RNAi Screen of DAF-16/FOXO Target Genes in C. elegans Links Pathogenesis and Dauer Formation
Source: PLoS One. 2010 Dec 31;5(12):e15902. doi: 10.1371/journal.pone.0015902 (PMC3013133; doi:10.1371/journal.pone.0015902)

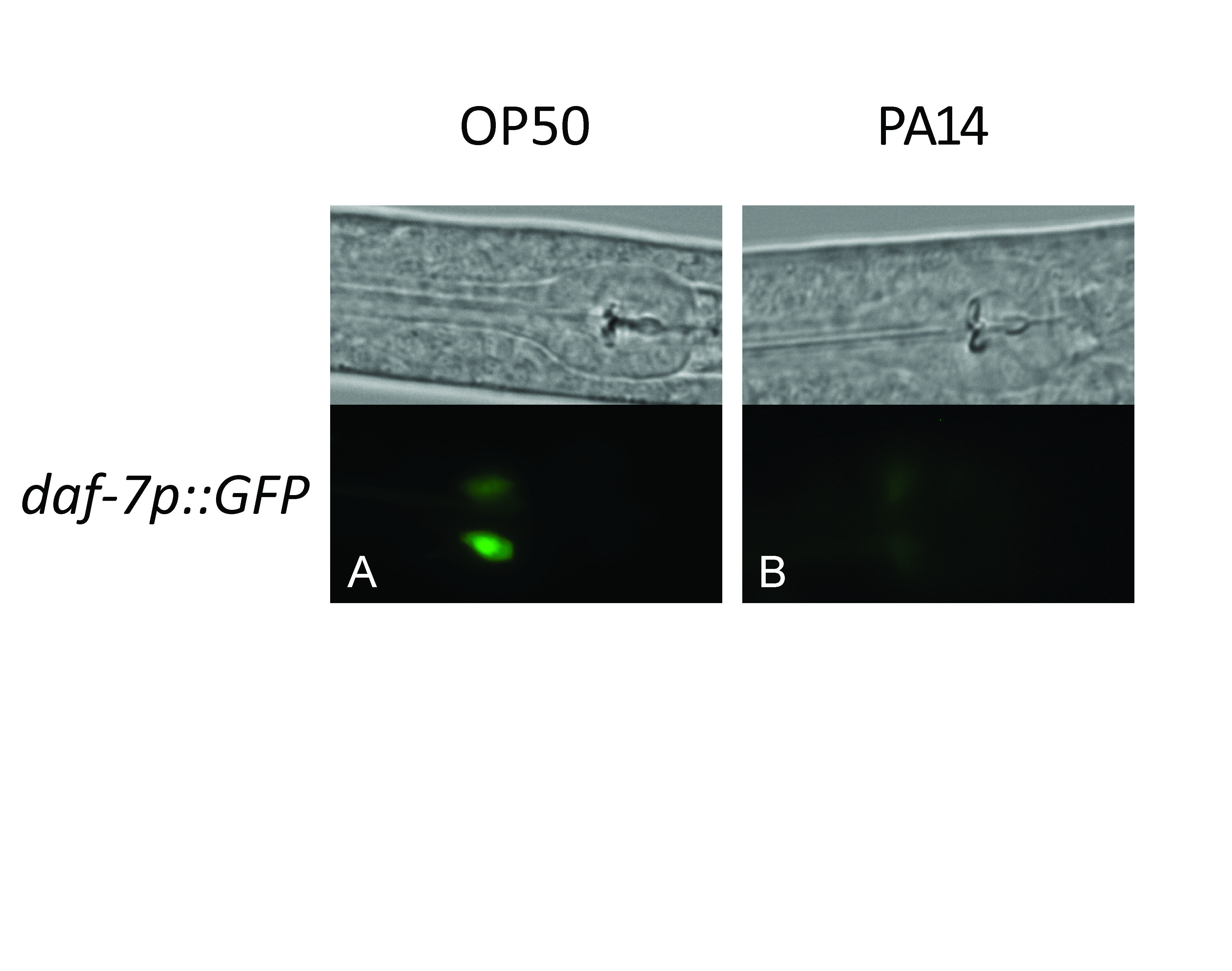

Supplement: Figure S1 — Reduction in daf-7 expression on PA14. In (A), the native GFP expression on the standard laboratory food E. coli OP50 from a daf-7 promoter driving expression of GFP. The expression of daf-7 is much reduced after are exposure to the strong pathogen PA14, as seen in (B). Images were taken with a 100× objective and 10× ocular lenses, eight hours after L2 larvae were transferred to either OP50 or PA14 from OP50 plates. (TIF) [file pone.0015902.s001.tif]
