# Supplementary material for: RNAi Screen of DAF-16/FOXO Target Genes in C. elegans Links Pathogenesis and Dauer Formation
Source: PLoS One. 2010 Dec 31;5(12):e15902. doi: 10.1371/journal.pone.0015902 (PMC3013133; doi:10.1371/journal.pone.0015902)
Supplement: Table S1 — Full list of target RNAi clones. (DOCX) [file pone.0015902.s002.docx]

**Table S1 - Full list of target RNAi clones**

| B0024.4 | C09G1.1 | C34H4.2 | F02A9.3 | F21D5.3 | F45C12.7 |
| --- | --- | --- | --- | --- | --- |
| B0024.6 | C09G4.5 | C35E7.1 | F02G3.1 | F21F8.7 | F45D3.2 |
| B0213.15 | C10C5.4 | C35E7.5 | F07F6.1 | F22D6.3 | F46B6.8 |
| B0281.3 | C10C5.5 | C36A4.9 | F07F6.5 | F22H10.3 | F46E10.1 |
| B0281.5 | C10G8.4 | C37A2.1 | F08A8.2 | F23A7.4 | F46F3.3 |
| B0365.6 | C12C8.2 | C39D10.7 | F08C6.2 | F23B2.12 | F46G10.3 |
| B0491.5 | C12D12.1 | C39E9.1 | F08D12.2 | F23F12.3 | F46G10.4 |
| B0496.4 | C12D5.9 | C41H7.7 | F08F1.4 | F23H11.7 | F47C10.2 |
| B0496.7 | C14A6.1 | C42C1.7 | F08F3.4 | F26E4.4 | F47G4.3 |
| B0511.8 | C14E2.2 | C43D7.4 | F08G5.6 | F27B3.5 | F48E3.4 |
| B0513.5 | C14F5.1 | C43D7.5 | F08H9.8 | F28B4.3 | F49C12.14 |
| B0554.6 | C15C6.3 | C44B12.1 | F08H9.9 | F28C1.3 | F49C12.7 |
| C01A2.2 | C15H9.7 | C44B7.5 | F09B9.1 | F28D1.4 | F49E12.1 |
| C01A2.3 | C15H9.9 | C44F1.2 | F09E10.1 | F28D1.5 | F49E12.2 |
| C01B7.1 | C16A3.10 | C46A5.5 | F09F3.9 | F28G4.1 | F49E12.9 |
| C01B7.3 | C16C10.5 | C46E10.3 | F09G8.8 | F29G9.1 | F49F1.1 |
| C01F1.2 | C16C4.4 | C46E10.4 | F10A3.9 | F31C3.6 | F49F1.5 |
| C03C10.2 | C16H3.2 | C46E10.7 | F10D11.1 | F31F7.1 | F52D2.6 |
| C03C10.5 | C17B7.1 | C47B2.6 | F10D7.5 | F32A5.3 | F52E1.1 |
| C03E10.6 | C17D12.2 | C47B2.8 | F10G2.3 | F32H5.1 | F52E1.5 |
| C03G6.15 | C17D12.6 | C47D12.6 | F11A5.10 | F34D10.4 | F53A9.8 |
| C04F5.1 | C17H12.6 | C48B4.1 | F11C1.3 | F35C5.9 | F53C11.1 |
| C04F6.1 | C17H12.8 | C48E7.2 | F12B6.2 | F35E12.10 | F53C3.2 |
| C05A9.1 | C18E3.9 | C49C3.9 | F13A7.11 | F35E12.5 | F53F4.8 |
| C05B5.1 | C18E9.5 | C50E3.12 | F13A7.9 | F35E12.7 | F53G12.5 |
| C05B5.2 | C18H7.4 | C52D10.7 | F13C5.4 | F35E12.8 | F53G2.6 |
| C05C10.4 | C18H7.6 | C52D10.9 | F13H6.3 | F35E12.9 | F53H4.2 |
| C05D12.2 | C18H9.6 | C52E4.1 | F14B4.3 | F35E2.2 | F54B11.3 |
| C05D2.7 | C24G6.6 | C53A3.2 | F14B6.3 | F35E8.11 | F54D10.8 |
| C05D2.8 | C25A8.4 | C53B7.2 | F14B8.3 | F35F10.10 | F54D5.4 |
| C05D9.8 | C25D7.5 | C54D1.2 | F14H3.12 | F36F2.1 | F54E2.1 |
| C05E11.5 | C25E10.1 | C54G4.6 | F15E11.1 | F36G9.12 | F54F11.2 |
| C05G5.6 | C25H3.9 | C56E6.1 | F15E11.12 | F36H5.8 | F54F2.2 |
| C06B3.3 | C27A7.1 | D1025.4 | F15E11.7 | F37F2.3 | F54F3.1 |
| C06E1.6 | C28F5.3 | D1054.10 | F15E11.9 | F38A1.10 | F54F7.2 |
| C07A9.4 | C29A12.3 | D1054.11 | F16H6.1 | F38A1.9 | F55B11.1 |
| C07B5.5 | C29F3.7 | D2013.7 | F17E5.1 | F40A3.7 | F55G11.2 |
| C07E3.9 | C29F7.3 | D2023.7 | F17E9.11 | F40F12.2 | F55G11.5 |
| C07G3.9 | C31A11.5 | D2045.6 | F18A1.7 | F40F12.3 | F55G11.8 |
| C07H6.5 | C32D5.5 | D2085.3 | F18E2.1 | F40F4.1 | F56A4.G |
| C08B11.4 | C32E8.11 | E02C12.6 | F18E9.5 | F40F4.2 | F56A4.j |
| C08B6.10 | C32H11.1 | E02C12.7 | F19C6.4 | F40F4.4 | F56A4.K |
| C08F11.11 | C32H11.10 | E02C12.8 | F19C7.1 | F41A4.1 | F56B6.1 |
| C08F11.12 | C32H11.12 | E03H4.11 | F19C7.2 | F41E7.4 | F56C9.7 |
| C08F11.8 | C32H11.13 | EGAP2.3 | F19C7.4 | F42G10.1 | F56D5.5 |
| C08F8.5 | C32H11.2 | F01D4.2 | F19C7.6 | F42G4.5 | F56F3.2 |
| C08H9.5 | C32H11.4 | F01D5.3 | F19F10.8 | F44D12.8 | F56G4.2 |
| C09D4.5 | C32H11.9 | F01D5.5 | F20C5.2 | F44E7.2 | F56G4.3 |
| C09F12.1 | C34H4.1 | F01G10.3 | F20H11.5 | F44G4.1 | F57C2.4 |
| F57F4.3 | K12H4.7 | T12D8.1 | Y119D3_451.B | Y71H10B.1 |  |
| F57F4.4 | M01F1.7 | T13B5.3 | Y119D3_457.C | Y75B8A.4 |  |
| F57F5.1 | M01H9.3 | T13F2.8 | Y14H12B.2 | ZC155.6 |  |
| F57G4.1 | M02F4.3 | T15B7.9 | Y17G7B.8 | ZC266.2 |  |
| F58A3.1 | M02F4.7 | T16A9.1 | Y17G9A.E | ZC302.2 |  |
| F58A4.3 | M03C11.5 | T16G1.4 | Y19D10A.j | ZC416.6 |  |
| F58A4.7 | M03F4.7 | T16G1.6 | Y19D10A.K | ZC443.5 |  |
| F58B3.9 | M04D8.1 | T16G12.1 | Y22F5A.4 | ZK105.F |  |
| F58B4.5 | M163.3 | T16G12.4 | Y37D8A.12 | ZK1127.10 |  |
| F58G1.4 | M28.8 | T16G12.7 | Y38H6C.1 | ZK1127.3 |  |
| F59A3.3 | M60.1 | T16H12.1 | Y38H6C.3 | ZK1193.2 |  |
| F59B1.2 | M7.2 | T16H12.8 | Y38H6C.5 | ZK1193.4 |  |
| F59C6.4 | R03G5.5 | T17A3.7 | Y39C12A.A | ZK1251.2 |  |
| F59C6.5 | R03G8.3 | T19C9.8 | Y39G10A_243.C | ZK1290.6 |  |
| F59D8.A | R03G8.6 | T19D12.4 | Y41D4B_7946.B | ZK1320.1 |  |
| F59D8.B | R05F9.10 | T20B3.12 | Y43C5B.2 | ZK177.8 |  |
| F59D8.C | R05F9.12 | T21C12.2 | Y43F4A.3 | ZK218.6 |  |
| F59D8.d | R07B1.10 | T21G5.2 | Y43F8B.9 | Zk218.8 |  |
| F59D8.E | R08E3.1 | T22G5.2 | Y43F8C.16 | ZK228.3 |  |
| F59D8.F | R09B5.3 | T24B8.3 | Y43F8C.2 | ZK228.4 |  |
| H04D03.1 | R09H10.5 | T24B8.5 | Y45F10A.2 | ZK484.2 |  |
| H12C20.2 | R09H3.3 | T25B6.2 | Y45F10C.2 | ZK546.13 |  |
| H13N06.6 | R102.4 | T25C12.3 | Y45F10C.4 | ZK546.17 |  |
| H19N07.1 | R107.8 | T25E12.6 | Y46C8_100.B | ZK6.10 |  |
| H20E11.1 | R10H10.3 | T27A10.4 | Y46C8_103.a | ZK6.7 |  |
| K01A2.5 | R13H4.3 | T28F2.5 | Y46C8_95.B | ZK688.6 |  |
| K01C8.5 | R193.D | VW02B12L.1 | Y46H3B.B | ZK757.1 |  |
| K01C8.6 | R53.4 | W01A11.4 | Y46H3C_14.c | ZK757.2 |  |
| K01G5.3 | T01D3.6 | W01B11.3 | Y47H10A.F | ZK813.2 |  |
| K02B12.2 | T03D3.1 | W02B12.1 | Y47H9C.1 | ZK896.2 |  |
| K02H11.2 | T03E6.7 | W02D9.6 | Y48A6B.6 | ZK896.5 |  |
| K04E7.2 | T03F6.1 | W02D9.7 | Y48A6B.7 | ZK896.7 |  |
| K04H4.6 | T05A10.1 | W02H3.1 | Y48A6C.1 | ZK896.8 |  |
| K05F1.10 | T05A8.5 | W03G1.7 | Y49E10.1 | ZK899.8 |  |
| K06A9.1 | T05D4.2 | W04A4.2 | Y49E10.8 |  |  |
| K06B9.4 | T05E7.1 | W04E12.6 | Y51A2D.13 |  |  |
| K06G5.1 | T06G6.11 | W04G3.6 | Y51A2D.4 |  |  |
| K07C6.5 | T07F10.1 | W05F2.2 | Y53F4C.J |  |  |
| K08C7.6 | T07H3.3 | W06B11.3 | Y55F3A_747.a |  |  |
| K08D8.5 | T08A9.8 | W07G4.2 | Y55F3A_748.b |  |  |
| K08D8.6 | T08G5.10 | W07G4.3 | Y56A3A.15 |  |  |
| K08E5.3 | T09E11.11 | W08D2.3 | Y57A10C.7 |  |  |
| K09D9.1 | T09E11.2 | W09C3.7 | Y57G11B.5 |  |  |
| K09D9.2 | T10B11.3 | Y106G6H.10 | Y59E1B.2 |  |  |
| K09F5.2 | T10C6.8 | Y106G6H.9 | Y62H9A.3 |  |  |
| K09H11.7 | T10G3.3 | Y116A8A.2 | Y62H9A.4 |  |  |
| K10D11.1 | T11F9.12 | Y116A8C.17 | Y62H9A.5 |  |  |
| K11B4.1 | T11F9.3 | Y116A8C.35 | Y62H9A.6 |  |  |
| K11D9.2 | T12B5.10 | Y116F11A.H | Y67D8A_380.C |  |  |
