# Supplementary material for: RNAi Screen of DAF-16/FOXO Target Genes in C. elegans Links Pathogenesis and Dauer Formation
Source: PLoS One. 2010 Dec 31;5(12):e15902. doi: 10.1371/journal.pone.0015902 (PMC3013133; doi:10.1371/journal.pone.0015902)
Supplement: Table S2 — Dauer and Non-dauer worm counts for positive genes. (DOCX) [file pone.0015902.s003.docx]

**Table S2 - Dauer and Non-dauer worm counts for positive genes.**

| Count 1 | | | | | |
| --- | --- | --- | --- | --- | --- |
| Gene | Non-dauer | Dauer larva | Total | Percent Dauer | χ2 p-value |
| GFP | 71 | 9 | 80 | 11.3% |  |
| *akt-1* | 88 | 58 | 146 | 39.7% | 1.3E-27 |
| *srh-100* | 42 | 28 | 70 | 40.0% | 2.7E-14 |
| *lase-1* | 104 | 61 | 165 | 37.0% | 1.4E-25 |
| F44D12.8 | 73 | 17 | 90 | 18.9% | 2.2E-02 |
| ZK896.5 | 53 | 19 | 72 | 26.4% | 4.8E-05 |
| *skr-8* | 80 | 29 | 109 | 26.6% | 3.9E-07 |
| *dct-5* | 16 | 10 | 26 | 38.5% | 1.1E-05 |
| F52E1.5 | 43 | 11 | 54 | 20.4% | 3.4E-02 |
| *cpr-1* | 37 | 24 | 61 | 39.3% | 3.8E-12 |
| Count 2 | | | | | |
| Gene | Non-dauer | Dauer larva | Total | Percent Dauer | χ2 p-value |
| GFP | 150 | 11 | 161 | 6.8% |  |
| *akt-1* | 14 | 32 | 46 | 69.6% | 8.3E-64 |
| *srh-100* | 35 | 50 | 85 | 58.8% | 1.7E-80 |
| *lase-1* | 9 | 26 | 35 | 74.3% | 2.4E-56 |
| F44D12.8 | 2 | 6 | 8 | 75.0% | 2.1E-14 |
| *cyp-35A3* | 8 | 34 | 42 | 81.0% | 8.1E-81 |
| C24G6.6 | 14 | 33 | 47 | 70.2% | 1.8E-66 |
| *dct-17* | 2 | 19 | 21 | 90.5% | 4.0E-52 |
| F35E12.9 | 49 | 31 | 80 | 38.8% | 1.1E-29 |
| F35E12.10 | 46 | 40 | 86 | 46.5% | 3.5E-48 |
| *dct-14* | 91 | 49 | 140 | 35.0% | 7.7E-40 |
| *clc-1* | 14 | 18 | 32 | 56.3% | 1.6E-28 |
| *unc-84* | 36 | 9 | 45 | 20.0% | 4.6E-04 |
| *ccb-1* | 72 | 30 | 102 | 29.4% | 1.6E-19 |
| F59B1.2 | 71 | 64 | 135 | 35.4% | 1.2E-28 |
| C53A3.2 | 69 | 25 | 94 | 47.4% | 6.5E-78 |
| E02C12.8 | 75 | 32 | 107 | 26.6% | 3.1E-14 |
| *lys-1* | 39 | 15 | 54 | 29.9% | 3.1E-21 |
| Count 3 | | | | | |
| Gene | Non-dauer | Dauer larva | Total | Percent Dauer | χ2 p-value |
| GFP | 107 | 2 | 109 | 1.8% |  |
| *akt-1* | 117 | 300 | 417 | 71.9% | 0 |
| ZK896.5 | 29 | 38 | 67 | 56.7% | 1.2E-245 |
| *skr-8* | 56 | 22 | 78 | 28.2% | 1.9E-67 |
| *cyp-35A3* | 119 | 16 | 135 | 11.9% | 4.2E-18 |
| C24G6.6 | 23 | 9 | 32 | 28.1% | 1.5E-28 |
| *dct-17* | 259 | 39 | 298 | 13.1% | 1.8E-47 |
| F35E12.9 | 93 | 13 | 106 | 12.3% | 1.2E-15 |
| F35E12.10 | 290 | 29 | 319 | 9.1% | 4.6E-22 |
| *dct-5* | 428 | 41 | 469 | 8.7% | 7.5E-29 |
| F59B1.2 | 135 | 53 | 188 | 28.2% | 1.1E-159 |
| C53A3.2 | 244 | 77 | 321 | 24.0% | 3.3E-192 |
| F52E1.5 | 78 | 8 | 86 | 9.3% | 2.5E-07 |
| E02C12.8 | 275 | 121 | 396 | 30.6% | 0 |
| *lys-1* | 121 | 30 | 151 | 19.9% | 3.1E-61 |
| *cpr-1* | 71 | 27 | 98 | 27.6% | 3.1E-80 |
| Count 4 | | | | | |
| Gene | Non-dauer | Dauer larva | Total | Percent Dauer | χ2 p-value |
| GFP | 633 | 37 | 670 | 5.5% |  |
| *akt-1* | 213 | 28 | 241 | 11.6% | 3.4E-05 |
| *srh-100* | 224 | 38 | 262 | 14.5% | 2.0E-10 |
| *lase-1* | 51 | 216 | 267 | 80.9% | 0 |
| F44D12.8 | 142 | 225 | 367 | 61.3% | 0 |
| ZK896.5 | 228 | 69 | 297 | 23.2% | 1.0E-40 |
| *skr-8* | 173 | 60 | 233 | 25.8% | 1.2E-41 |
| *cyp-35A3* | 98 | 14 | 112 | 12.5% | 1.2E-03 |
| C24G6.6 | 205 | 45 | 250 | 18.0% | 5.8E-18 |
| *dct-17* | 305 | 50 | 355 | 14.1% | 1.6E-12 |
| F35E12.9 | 51 | 39 | 90 | 43.3% | 1.4E-55 |
| F35E12.10 | 215 | 25 | 240 | 10.4% | 9.0E-04 |
| *dct-14* | 108 | 49 | 157 | 31.2% | 4.3E-45 |
| *clc-1* | 38 | 42 | 80 | 52.5% | 1.4E-75 |
| *unc-84* | 147 | 114 | 261 | 43.7% | 2.1E-160 |
| *ccb-1* | 223 | 44 | 267 | 16.5% | 4.6E-15 |
| Count 5 | | | | | |
| Gene | Non-dauer | Dauer larva | Total | Percent Dauer | χ2 p-value |
| GFP | 446 | 6 | 452 | 1.3% |  |
| *akt-1* | 291 | 51 | 342 | 14.9% | 8.4E-107 |
| F59B1.2 | 356 | 29 | 385 | 7.5% | 2.0E-26 |
| C53A3.2 | 390 | 37 | 427 | 8.7% | 4.6E-40 |
| F52E1.5 | 595 | 50 | 645 | 7.8% | 4.1E-46 |
| E02C12.8 | 399 | 25 | 424 | 5.9% | 2.0E-16 |
| *lys-1* | 333 | 27 | 360 | 7.5% | 1.4E-24 |
| *cpr-1* | 196 | 78 | 274 | 28.5% | 0 |
| Count 6 | | | | | |
| Gene | Non-dauer | Dauer larva | Total | Percent Dauer | χ2 p-value |
| GFP | 209 | 18 | 227 | 7.9% |  |
| *akt-1* | 227 | 53 | 280 | 18.9% | 9.7E-12 |
| *dct-14* | 141 | 71 | 212 | 33.5% | 3.6E-43 |
| *clc-1* | 177 | 89 | 266 | 33.5% | 1.4E-53 |
| *unc-84* | 99 | 25 | 124 | 20.2% | 4.6E-07 |
| *ccb-1* | 188 | 74 | 262 | 28.2% | 4.5E-34 |
| *dct-5* | 150 | 22 | 172 | 12.8% | 1.8E-02 |
| Count 7 | | | | | |
| Gene | Non-dauer | Dauer larva | Total | Percent Dauer | χ2 p-value |
| GFP | 217 | 10 | 227 | 4.4% |  |
| *akt-1* | 18 | 71 | 89 | 79.8% | 4.6E-263 |
| F44D12.8 | 82 | 21 | 103 | 20.4% | 2.7E-15 |
| *dct-17* | 83 | 33 | 116 | 28.4% | 1.7E-36 |
| *dct-5* | 117 | 22 | 139 | 15.8% | 5.3E-11 |
